# KDM4 Involvement in Breast Cancer and Possible Therapeutic Approaches

**DOI:** 10.3389/fonc.2021.750315

**Published:** 2021-10-28

**Authors:** Benluvankar Varghese, Nunzio Del Gaudio, Gilda Cobellis, Lucia Altucci, Angela Nebbioso

**Affiliations:** ^1^ Department of Precision Medicine, University of Campania Luigi Vanvitelli, Napoli, Italy; ^2^ Biogem Institute of Molecular Biology and Genetics, Ariano Irpino, Italy; ^3^ Saint Camillus International University of Health and Medical Sciences, Rome, Italy

**Keywords:** epigenetics, histone demethylation, KDM4 inhibitors, JMJD2, KDM4

## Abstract

Breast cancer (BC) is the second leading cause of cancer death in women, although recent scientific and technological achievements have led to significant improvements in progression-free disease and overall survival of patients. Genetic mutations and epigenetic modifications play a critical role in deregulating gene expression, leading to uncontrolled cell proliferation and cancer progression. Aberrant histone modifications are one of the most frequent epigenetic mechanisms occurring in cancer. In particular, methylation and demethylation of specific lysine residues alter gene accessibility *via* histone lysine methyltransferases (KMTs) and histone lysine demethylases (KDMs). The KDM family includes more than 30 members, grouped into six subfamilies and two classes based on their sequency homology and catalytic mechanisms, respectively. Specifically, the *KDM4* gene family comprises six members, *KDM4A-F*, which are associated with oncogene activation, tumor suppressor silencing, alteration of hormone receptor downstream signaling, and chromosomal instability. Blocking the activity of KDM4 enzymes renders them “druggable” targets with therapeutic effects. Several KDM4 inhibitors have already been identified as anticancer drugs *in vitro* in BC cells. However, no KDM4 inhibitors have as yet entered clinical trials due to a number of issues, including structural similarities between KDM4 members and conservation of the active domain, which makes the discovery of selective inhibitors challenging. Here, we summarize our current knowledge of the molecular functions of KDM4 members in BC, describe currently available KDM4 inhibitors, and discuss their potential use in BC therapy.

## Introduction

Breast cancer (BC) is the second leading cause of cancer death in women worldwide with a 0.5% increase in incidence rate per year. Advances in diagnosis and treatment in 64% of BC cases at earlier stages has increased 5-year survival to 99% (National Breast Cancer Foundation).

Much is known about oncogenes, tumor suppressors, and DNA repair genes, which play a role in breast tumorigenesis, promoting aberrant cell growth and/or mismatch error repair (1, 2). Research on molecular hallmarks of BC has identified several diagnostic markers including: i) immunohistochemical markers, such as estrogen receptor (ER), progesterone receptor (PR), and human epidermal growth factor receptor 2 (HER2); ii) genetic markers, such as *BRCA1*, *BRCA2*, and *PIK3CA* mutations; iii) immunomarkers, such as programmed death-ligand 1 (PD-L1) and tumor infiltrating lymphocytes; iv) proliferation markers, such as Ki-67. All of these have significantly changed the prediction of prognosis and therapy decisions (3).

The Cancer Genome Atlas classifies BC into five different subtypes: normal-like, luminal A, luminal B, HER2-positive (HER2^+^), and basal-like. Luminal A and B tumors are ER- and PR-positive (ER^+^PR^+^), while the HER2^+^ and basal-like subtypes are hormone-independent (ER^-^PR^-^) and positive for high levels of Ki-67, showing the worst prognosis (4).

Current therapeutic strategies for BC are based on tumor heterogeneity associated with different histotypes and specific molecular profiles: ER^+^ and PR^+^ patients are treated with hormonal therapy, HER2^+^ patients with anti-HER2 therapy, and *BRCA* mutation carriers with poly (ADP-ribose) polymerase (PARP) inhibitors plus adjuvant therapies (chemotherapy, immunotherapy, and radiation therapy) (5).

Despite advancements in our knowledge of BC biology as well as intense disease prevention programs and therapies able to block tumor progression, the incidence of BC continues to rise. High-throughput analysis reveals a massive transcriptional deregulation in BC, for which a tight interplay between genetic and epigenetic factors has been hypothesized. Progressive dedifferentiation of cell identity to a progenitor-like state due to increased cell plasticity is observed in the early phase of cancer formation, whereas epigenetic modifications support oncogenic progression (6).

Epigenetic alterations such as DNA methylation and reversible histone modifications (methylation, acetylation, ubiquitination) alter gene accessibility, resulting in aberrant gene expression.

A promising opportunity to rewind cell fate comes from epigenetic-based therapies, which make use of small molecule drugs (epidrugs) able to interfere with the activity of epigenetic regulators and thus correct cancer-associated chromatin states (7). Following confirmation of the efficacy of epidrug-based therapies in oncology by several *in vitro* studies, many epidrugs have moved to clinical trials for different cancer types (8), and some have been clinically approved by the US Food and Drug Administration (9).

Eukaryotic chromatin is organized in active euchromatin and inactive heterochromatin and the histone methylations define these two interchangeable functional states. Histone lysine methylation is regulated by methyltransferases (KMTs) and demethylases (KDMs) (10). KDMs are classified into two groups: i) the KDM1 or LSD1 family, dependent on flavin adenine dinucleotide (FAD) and ii) the JmjC family, dependent on 2-oxoglutarate (2-OG) for their demethylase activity. JmjC domain-containing KDMs form the larger KDM class with 20 members grouped into five subfamilies (KDM2/7, KDM3, KDM4, KDM5, and KDM6), and their deregulation is associated with cancer, including BC (11).

**Figure 1:**
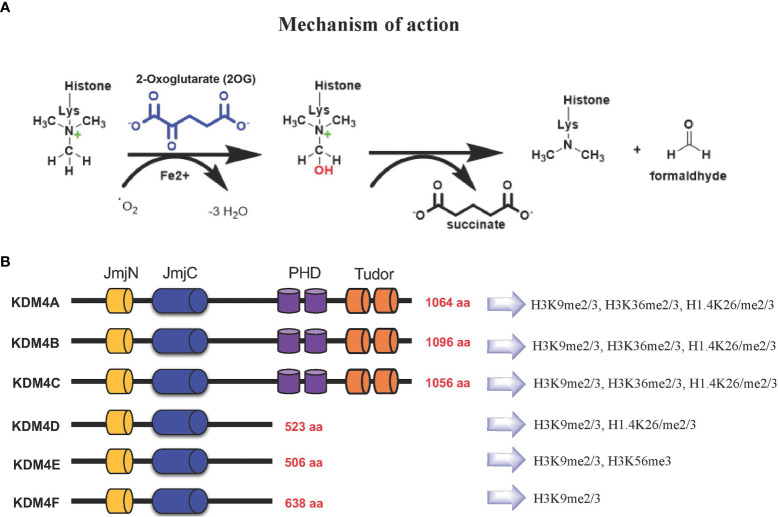
**(A)** KDM4s: mechanism of demethylation; **(B)** KDM4s have conserved JmjN and JmjC domains, while substrate recognition domains such as PHD and Tudor are present only in KDM4A-C. Histone targets of KDM4 family members are shown on the right.

Based on their catalytic activity, KDM4 subfamily members catalyze N-methyl-lysine demethylation by removing mono-, di-, and trimethyl marks *via* an oxidative mechanism. KDM4 uses 2-OG and O_2_ as cosubstrates, Fe(II) as a cofactor for the enzymatic oxygenase reaction (**Figure 1A**). This activity contributes to the control of gene expression in a context-dependent manner, either by influencing the compaction of chromatin or through regulation of signaling pathways and recruitment of other protein complexes. The most frequent modifications occur on H3K4, H3K36 and H3K79 associated with gene activation, whereas H3K9, H3K27, H4K20 and H3K56 associated with gene silencing (11–13).

The KDM4 (JHDM3/JMJD2) subfamily is highly conserved (14, 15). In humans, this subfamily comprises *KDM4A*, *KDM4B*, *KDM4C*, and *KDM4D* genes, with *KDM4E* and *KDM4F* considered as pseudogenes, although a partial catalytic activity is reported (16) (**Figure 1B**).

KDM4A-C enzymes have five different domains: JmjN, JmjC, tandem PHD, Tudor, and F-box, whereas KDM4D-F lack PHD and Tudor domains (**Figure 1B**) (17). The stability and catalytic activity of KDM4s depends on the interaction between JmjN and JmjC domains, and their structural integrity maintains overall protein stability (18). The crystal structure of the KDM4A Tudor domain revealed it as histone reader, identifying methylated lysine residues at histone H3 and H4 tails; the function of the PHD domain is still unclear, although in other PHD-containing proteins this domain is able to bind modified and unmodified histone residues (19).

Concerning KDM4 mRNA levels, they are tightly regulated to guarantee proper biological processes (20). Next-generation sequencing in normal tissues revealed that KDM4A/B/C are broadly expressed in most tissues, although at different levels. They share more than 50% protein sequence identity, however the variations in expression levels suggest that these proteins have not-overlapping functions, as evidenced also by single/double knockout mouse models, that were viable and showed no evident abnormalities. Cell-specificity is thus guaranteed by specific interaction with regulatory factors. For instance, the control of KDM4A expression rely on ubiquitin-proteasome pathway through FBX022, a key regulator of histone methylation. This evidence suggests that posttranslational modifications of KDM4A regulates its abundance, conferring it the cell/tissue-specificity (21). Other studies suggested that KDM4s have peculiar cell-type functions. Heart-specific KDM4A conditional knockout showed cardiac hypertrophy and no compensatory effect has been observed (22).

**Figure 2:**
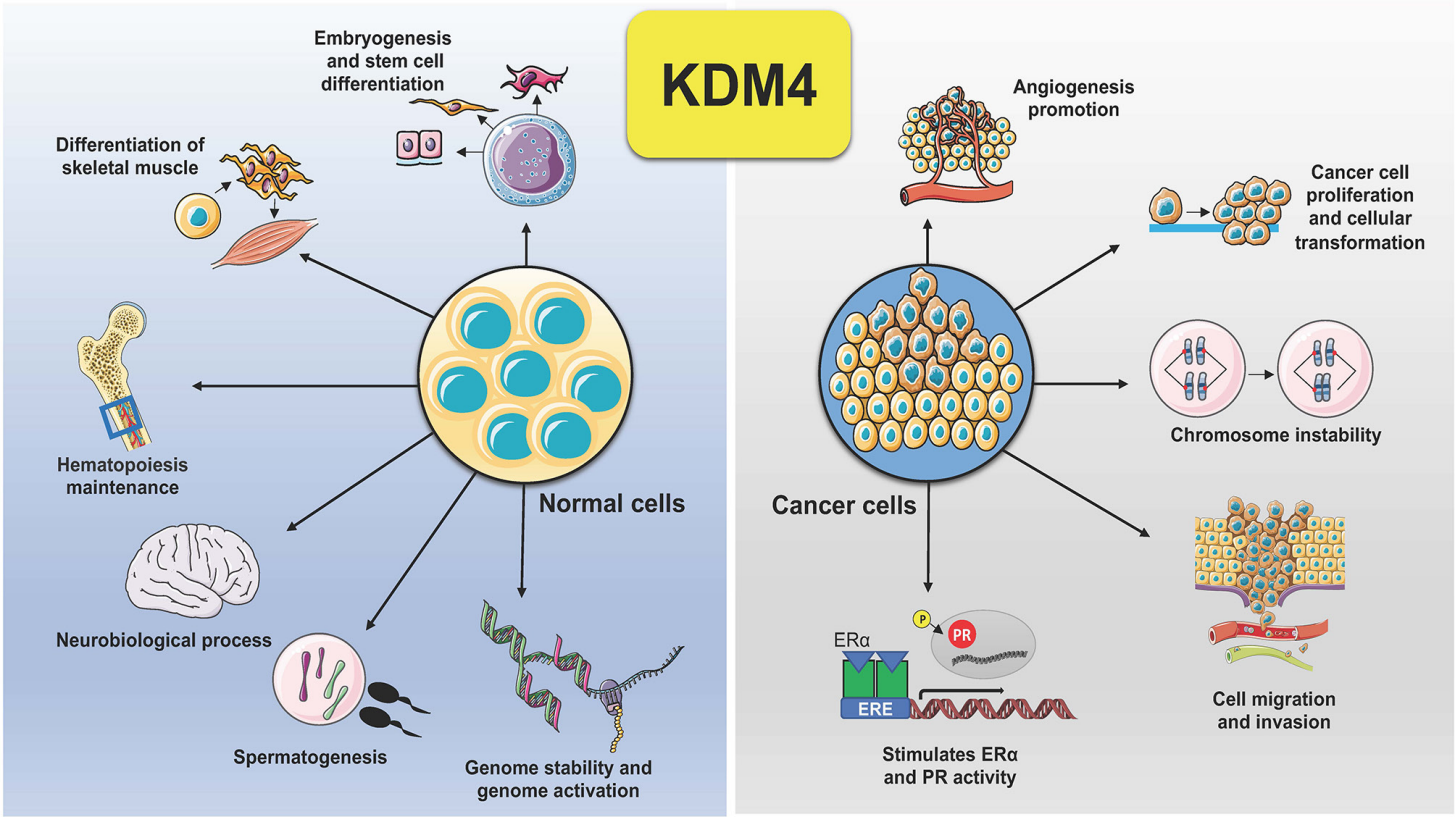
Schematic representation of KDM4 functions in normal and cancer cells. Deregulation of KDM4 promotes cancer cell proliferation, migration, and invasion, angiogenesis, chromosome instability, and stimulation of ER and PR activity.

KDM4 subfamily members control different biological functions, to ensure proliferation, differentiation, migration and adhesion (23), as well as regulation of transcription (24) and genome stability (25) (**Figure 2**). In embryonic stem cells (ESCs), KDM4 proteins (4B and 4C) control stem cell identity by interacting with the pluripotency factors such as Sox2, Oct4, c-Myc, and Klf4, but also by modulating, alone or in combination, gene expression during the differentiation program (26). In addition, depletion of KDM4C in ESCs causes downregulation of *Myc* and *Klf4* genes associated with cell proliferation during early embryo stage, leading to developmental defects (27).

KDM4s are also involved in cell differentiation: KDM4B promotes osteogenic differentiation of human mesenchymal stem cells, activating expression of *DLX* genes by removing trimethyl groups from H3K9me3 marks (28, 29), whereas depletion of KDM4B reduces osteogenic differentiation *via DLX* gene suppression (28).

Downregulation of *Taf1b* and *Nom1* in hematopoietic stem cells was observed due to accumulation of H3K9me3 on their transcription start sites (30), following knockout of KDM4A/B/C *in vivo* resulting in aberrant differentiation.

KDM4A was found to play an important role in skeletal muscle differentiation (31), while KDM4B deletion in mice leads to neurodevelopmental disorders and defects in spinal maturation (32). KDM4A knockdown or inhibition decreases leukocyte adhesion and transmigration in inflammatory response, by modulating expression of vascular adhesion proteins (ICAM1 and VCAM1) in cerebral microvessels (33). KDM4A/D play a role in female fertility (34) and in spermatogenesis, respectively (35). KDM4D was recently reported to maintain genome stability by facilitating double-strand DNA damage repair mechanisms in a PARP1-dependent manner; specifically, the interaction between KDM4D and RNA seems to be essential for chromatin localization and efficient demethylation of trimethyl H3K9 (36).

Concerning breast tissue, KDM4B is important for transcriptional regulation and development of mammary gland. Deletion of KDM4B in mammary epithelium produces immature mammary gland development in female mice (37). KDM4B is also involved in ER signaling cascade and is required for ER-mediated gene transcription, essential for normal development of ovarian follicles, luteal function, and ovulation (38–40). In summary, these findings revealed that KDM4B plays a critical role in regulation of transcriptional program in the mammary gland.

Dysregulation of KDM4s is behind several hallmarks of cancer (**Figure 2**). Tumorigenesis is a complex adaptive process that involves alterations in different cellular functions, as proliferation, differentiation, adaptation to altered microenvironment, many of them controlled by KDM4s, found overexpressed in various human cancers, sustaining tumor progression and acting as oncoproteins (11, 41).

Thus, KDM4s have emerged as a druggable targets in cancer to restore cell homeostasis by erasing inappropriate histone modifications distributed across the genome that are responsible of cell transformation. Although the drug discovery rationale is straightforward, the efficacy of KDM4 inhibitors identified to date is limited, mainly due to their lack of selectivity and/or specificity to the different KDM4 isoforms (42).

High expression levels of KDM4A were observed in squamous cell carcinoma as well as in ovarian and prostate cancer, where it is highly associated with chromosomal instability (43). KDM4A/C/D bind androgen receptor (AR) *in vitro* and *in vivo*, resulting in tumor cell proliferation through demethylation of H3K9me3 in AR target genes, stimulating AR-dependent transcription in combination with KDM1A (44, 45).

KDM4A is also directly involved in upregulation of the lung cancer-associated genes *CXCL5*, *ADAM12*, and *JAG1*, involved in angiogenesis promotion, tumor cell growth, and cell proliferation (46–50). Overexpression of KDM4C was found in non-small cell lung carcinoma (51) and osteosarcoma (52), where upregulation of fibroblast growth factor 2, promoted by KDM4B/C modulates cell migration, invasion, and proliferation in osteosarcoma metastasis (52).

Demethylation of H3K9 marks by KDM4D is involved in tumor necrosis factor α activation, associated with tumorigenesis and inflammatory response (53). KDM4D stimulates p53-dependent gene expression and acts as a pro-oncogenic factor, specifically on AR target genes in prostate and colon cancer cell growth (54). Further, KDM4A reduces activity of p53 pathway through inhibition of Ras-mediated chromodomain-helicase-DNA-binding protein 5 (CHD5) induction, blocking senescence and thereby promoting cell transformation (55).

In ovarian cancer, reduced levels of KDM4B led to an increase in H3K9me3 in the promoter regions of genes such as *PDGFB*, *LCN2*, and *LOXL*2, suppressing cell migration, invasion, and formation of spheroids *in vitro* (56). In gastrointestinal tumors, KDM4D promotes cancer progression by directly interacting with hypoxia-inducible factor (HIF) 1β gene and activating its expression *via* H3K9me3 demethylation of the vascular endothelial growth factor A promoter region (57). KDM4B expression was found to be activated by *HIF* genes, promoting cancer cell survival in a hypoxic setting (58–60).

In conclusion, KDM4s exert their effect mainly by altering the chromatin state and therefore the expression of genes involved in physiological functions that, when disrupted, cancer occurs.

## Functional Role of KDM4s in Breast Cancer

**Figure 3:**
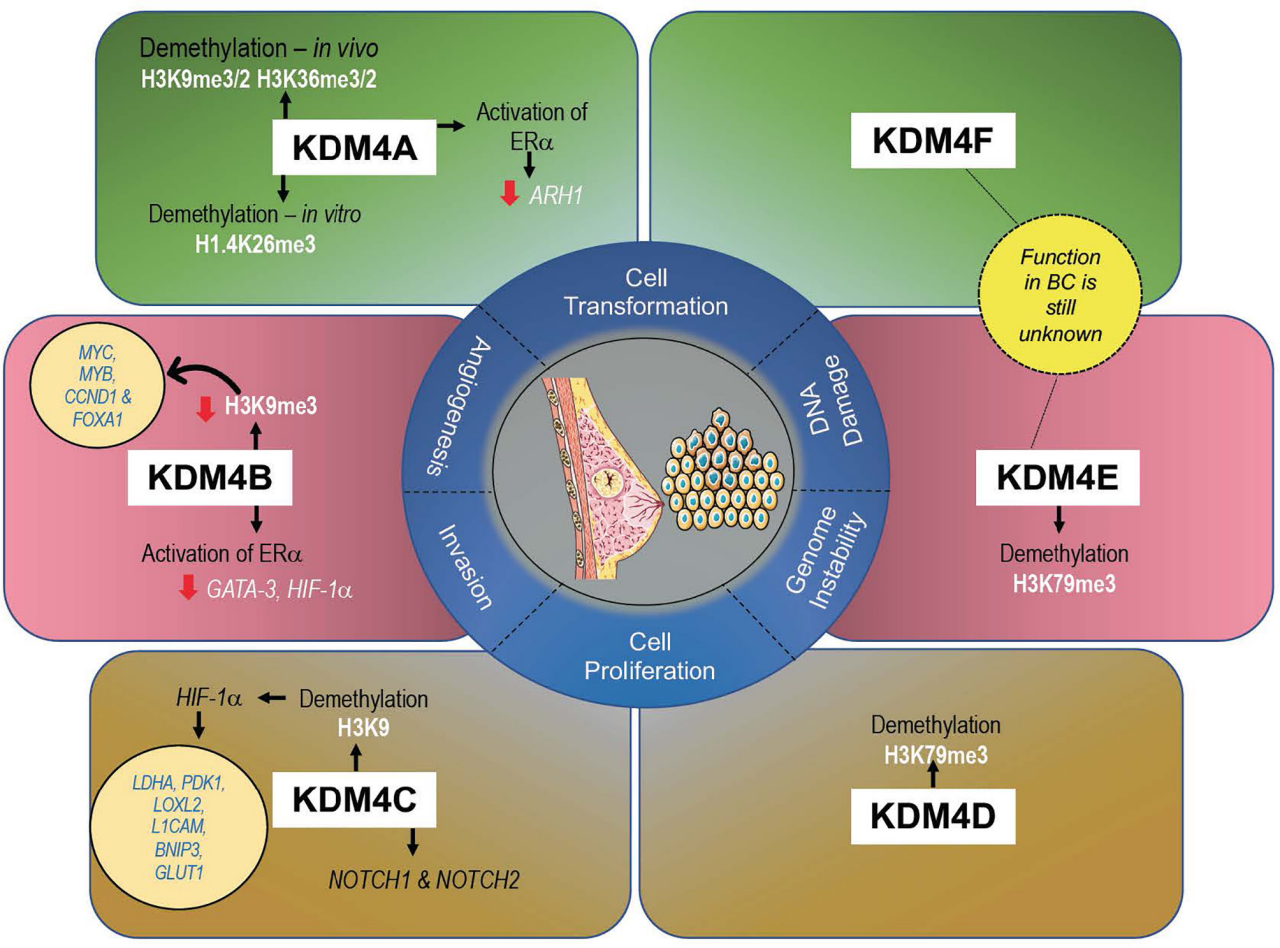
Expression levels of each KDM4 and its histone marks associated with BC progression.

KDM4s are responsible in controlling development and proliferation of mammary gland (61), and their altered expression (mainly gene amplification) can promote cell transformation, migration, and invasion, all hallmarks of tumorigenesis in BC (47) (**Figure 3**). A recent meta-analysis of KDM4 gene expression in BC subtypes identified overexpression of KDM4A/D in basal-like BC, whereas KDM4B was predominantly expressed in ER^+^ luminal-type BC (61).

The development of potential KDM4 inhibitors with high selectivity in different BC subtypes therefore remains a major challenge. To address this issue, a better understanding of the molecular mechanisms of KDM4s as well as their specific target sites is urgently required to develop new treatments targeting molecular pathways crucial for BC progression.

### KDM4A in BC

KDM4A mainly demethylates H3K9me2/me3 and, at a lower rate, H3K36me2/me3 *in vivo* and H1.4K26me3 *in vitro* (17), promoting chromatin decompaction. *Via* histone deacetylase (HDAC) and p53 association, KDM4A may repress gene expression (62, 63).

Overexpression of KDM4A was observed in 60% of BC tissue at both mRNA and protein level (64). KDM4A overexpression leads to upregulation of estrogen-dependent genes, whereas depletion of KDM4A decreases transcription of ERα target genes, such as *JUN* and *CCND1*, promoting cell growth arrest. Taken together, these interconnections suggest that KDM4A promotes BC growth *via* hormone-dependent and -independent mechanisms (65). KDM4A overexpression was also found to contribute to BC growth through downregulation of the tumor suppressor gene ADP-ribosylarginine hydrolase 1 (ARH1), highly expressed in normal breast tissue (66). Furthermore, downregulation of the ubiquitous transcription factor Sp1 was reported in highly invasive and in advanced stages of BC, showing a clear correlation with the TNM staging system, confirmed by KDM4A overexpression (67). *In vitro*, knockdown of KDM4A in MCF-7 cells blocks JUN expression, inhibiting invasion, migration, and tumor formation (68–70). In these cells, expression levels of KDM4A were also found to be modulated by hsa-mir-23a-3p, hsa-mir-23b-3p, and hsa-mir-137. Inhibition of these microRNAs enhances KDM4A levels, with a consequent increase in some drug-resistant genes such as CDC28 protein kinase regulatory subunit 1B (*CKS1B*) (71), contributing to the outgrowth of chemoresistant cells (68).

*CHD5*, a tumor suppressor gene, is under the control of KDM4A, whose silencing restores *CHD5* expression by decreasing H3K36me2/me3 histone marks in its locus (72).

### KDM4B in BC

KDM4B is similar to KDM4A in structure and enzymatic activity, demethylating both H3K9 and H3K36. Unlike KDM4A/C, KDM4B acts as a monomer and not as a homodimer or heterodimer (73).

KDM4B is a key regulator of estrogen signaling cascade, and its depletion attenuates BC growth both *in vitro* and *in vivo* (37, 40). Noteworthy, *KDM4B* is itself an ER-responsive gene (58). Taken together, these findings suggest a positive feedback mechanism between KDM4 and ER whereby estrogen-induced KDM4 expression in turn coregulates and, unexpectedly, upregulates ER-target genes, sustaining BC growth. KDM4B is required in ER-mediated gene transcription essential not only in mammary gland, but also in ovarian follicles, suggesting a possible correlation of KDM4B between these gynecological cancers.

H3K4 methylation and H3K9 demethylation are coordinated by binding of KDM4B/mixed-lineage leukemia 2 (MLL2 or KMT2D) complex, with ERα driving ERα-dependent transcription (74). Some studies report the interaction of KDM4B/ERα with SWI/SNF-B chromatin complex, regulating numerous genes involved in resistance and invasiveness of BC (37). Decreased levels of H3K9me3, corresponding to overexpression of KDM4B, facilitate transcription of ER-responsive genes such as *MYC*, *MYB*, *CCND1* (37), and *FOXA1* (40). GATA-3 is a transcription factor highly expressed in luminal A-type BC and is associated with ER expression. The demethylation process mediated by KDM4B is fundamental for activation of ER by GATA-3, whereas downregulation of KDM4B levels induces H3K9 methylation and a reduction in GATA-3 binding on ER promoter, suppressing ER targets (40). Moreover, ERα regulates expression of KDM4B through HIF-1α, promoting its expression in a feed-forward regulatory circuit (58).

Several ER coregulated genes are primed to activate gene expression upon histone modifications induced by KDM4 proteins. One example is the KDM3A/KDM4B/FOXA1 complex, which leads to an increase in pro-proliferative and ERα-dependent gene expression and dual knockdown of KDM4A and KDM4B, strongly inhibiting ERα activity and blocking cell proliferation (75).

Additionally, high KDM4B-mediated demethylation levels of H3K9 were found on the promoter of long interspersed nuclear element-1, increasing its expression and improving the effectiveness of retrotransposition (76). A direct correlation was found between KDM4B expression and the absence of H3K9me3 in pericentromeric regions, suggesting the involvement of this enzyme in chromosomal instability and aneuploidy cell formation (77).

Interestingly, KDM4B also plays a role at cytoplasmic level, where it regulates the unfolded protein response (UPR) pathway through direct interaction with eukaryotic initiation factor 2α (eIF2). UPR is commonly hyperactivated as result of severe and prolonged cellular stress, triggering cell death. Inhibiting the association between KDM4B and eIF2 also allows activation of UPR cell death pathway in triple-negative breast cancer (TNBC), deficient in PTEN, and therefore increases responsiveness to therapy with PI3K-AKT inhibitors (78).

Selective estrogen receptor modulators (SERMs) are beneficial in treating premenopausal ER-positive BC resistant to tamoxifen. However, no effect was obtained in tumors where *Fbxo22* gene is low expressed as Fbxo22 ubiquitinates tamoxifen-bound KDM4B (79), resulting in KDM4B overexpression and poor prognosis.

### KDM4C in BC

KDM4C (also known as GASC1) is amplified in many cancers including BC, mainly in basal-like and in ER^-^ and PR^-^ subtypes (80), making this enzyme a negative prognostic marker (81, 82). KDM4C overexpression is mediated by gene amplification of 9p24 chromosomal region, which contains several candidate tumor genes, including *KDM4C*.

KDM4C regulates expression of genes involved in stem cell self-renewal and induces phenotypic changes in cancer cells. However, despite its involvement in tumor development, proliferation, and aggression, very little is known about this enzyme compared to KDM4A/B. In MCF-10A cells, the expression of KDM4C induces a transformed phenotype (80). KDM4C upregulates many genes responsible for cell growth, migration, and metastasis and interacts with HIF-1α, mediating KDM4C recruitment on hypoxia-inducible genes and demethylation of H3K9 on metabolic genes, such as *LDHA*, *PDK1*, *LOXL2*, *L1CAM*, *BNIP3*, and *GLUT1*. The physical interaction of these two proteins is a critical epigenetic mechanism, given that HIF-1α involvement in BC is responsible for an aggressive phenotype, characterized by metastasis progression and resistance to drug therapy (83).

A D396N polymorphism found in the caspase-3 cleavage site of *KDM4C* in BC cells and contributes to drug resistance, indicating the involvement of KDM4C in BC-resistant progression (81).

Unlike KDM4A, KDM4C is recruited to mitotic chromosomes, modulating correct chromosomal stability and gene expression. This suggests that total inhibition of the enzyme in TNBC should induce a reduction in cell multiplication (84) and an increase in γ-H2AX, a marker of DNA damage (81). Through modulation of steroid receptor co-activator 1 (SRC-1), KDM4C also regulates CD24 and the apoptotic protein PAWR. In endocrine-resistant BC cell lines, SRC-1/KDM4C complex together with JUN mediates transcriptional repression of these two oncogenic proteins (85).

Despite evidence that KDM4C silencing or inhibition may represent an effective epigenetic therapy in BC treatment, a study conducted on 355 patients with invasive BC found that KDM4C was negatively associated with the development of a more aggressive BC histopathological type (grade II/III, ductal-type, PR^-^, and ER^-^). Women with KDM4C-positive tumors responded better to radiation therapy and hormone treatment (82).

### KDM4D/E/F in BC

Unlike other subfamily members, KDM4D has JmjN and JmjC domains encoding only a small peptide protein. KDM4D potentially regulates H3K79me3, suggesting its involvement in DNA repair, telomeric silencing regulation, cellular development, transcriptional regulation, and cell cycle checkpoints (86). The role of KDM4D in cancer is relatively less studied than that of other KDM4s. A recent study reported that KDM4D was significantly overexpressed in basal-like BC, with an amplification frequency of 3.6%, and was found ubiquitously expressed in ER^+^, MCF-10A, and basal-like cell lines (61).

The catalytic domain of KDM4E was found to demethylate H3K9me3/me2 regulated by the availability of O_2_ in an *in vitro* assay (87).

The expression of *KDM4E*/*F* in BC is still unknown. Further studies on these genes may unveil their potential role in BC and in other cancers.

### KDM4 Inhibitors

**Figure 4:**
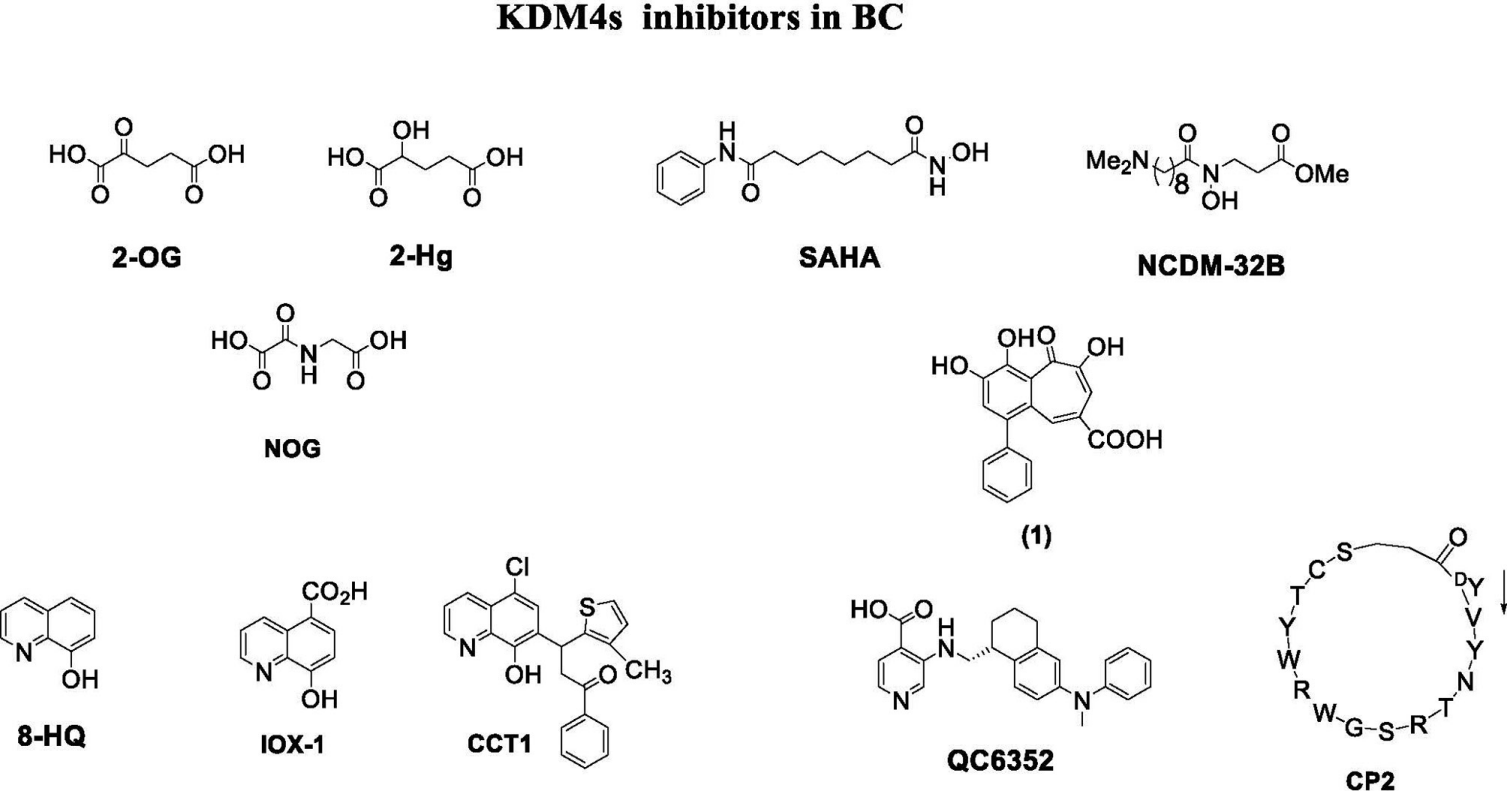
KDM4 inhibitors in BC.

Depending on their mechanism of action, KDM4 inhibitors are divided into different classes: 2-OG cofactor mimics, metal cofactor disruptors, histone substrate-competitive inhibitors, and natural and peptide inhibitors (**Figure 4**) (88).

Cofactor mimics are metal-chelated inhibitors that competitively bind Fe(II) molecules of the catalytic site of KDM4 members, blocking their enzymatic activity. Cancer cells are able to reprogram their metabolism to support the increased energy demand required for cell survival and rapid proliferation. Metabolic disruption can alter KDM4 activity by modifying the availability of the required cofactor, 2-OG. Therefore, the intermediates of the tricarboxylic acid (TCA) cycle can inhibit KDM activity. The first identified KDM4 inhibitors were the natural molecules fumarate and succinate, which act as competitive antagonists for 2-OG (89).

Among 2-OG analogs, the oxalic acid-derivative N-oxalylglycine (NOG: IC_50_ = 78 µM), pyridine dicarboxylic acid (PCA: IC_50_ = 1.4 µM), and 8-hydroxyquinoline (8-HQ: IC_50_ = 0.6 µM) showed antiproliferative activity (42).

The hydroxamate-based 2-OG analog NCDM-32B was identified as a good inhibitor of KDM4 subfamily members, and its therapeutic potential was investigated in basal-like BC (61). In enzymatic assays, NCDM-32B displayed IC_50_ values of 3.0 µM for KDM4A and 1.0 µM for KDM4C. Treatment with NCDM-32B in BC cell lines induced a global increase in H3K9me3/me2 marks, and microarray Gene Ontology analysis of differentially expressed genes revealed pathways that control cell proliferation, growth, DNA replication, and DNA repair. Of note, the compound suppressed the expression of oncogenes, such as the *MET* proto-oncogene, as well as genes involved in cell cycle regulation including *CDC26* and *CDK6.* These data suggest that NCDM-32B may be a regulator of different cell growth and transformation pathways activated in BC (61).

The orally available KDM4 inhibitor QC6352 has an IC_50_ value of 0.104 µM for KDM4A, 0.056 µM for KDM4B, and 0.035 µM for KDM4C (90). This molecule showed a strong capability to inhibit proliferation, sphere formation, and xenograft tumor formation of BC stem-like cells derived from tissue of TNBC patients after neoadjuvant chemotherapy. *Via* H3K9me3 induction, QC6352 inhibited expression of epidermal growth factor receptor, a pivotal gene in therapy resistance mechanisms in TNBC (91).

A very recent study characterized TACH101 as a first-in-class pan inhibitor of KDM4s, with promising pharmacological applicability. Surprisingly, the compound displayed potent inhibitory activity on four KDM4 isoforms (A-D) with IC_50_ values below 0.100 µM. Furthermore, it increased H3K36me3 levels and induced apoptosis in human esophageal cancer, TNBC, and colorectal cancer cell lines. *In vivo*, TACH101 showed 100% tumor growth inhibition in BC xenograft models, reducing tumor-initiating cell frequencies by 4.4-fold, and exhibited good oral availability. However, further preclinical studies are required to drive progression of the compound to clinical trials (92).

Another interesting study reported that KDM4 inhibitors such as A1 (CGC00247751), B3 (NCGC00244536), and I9 (NCGC00247743) repress the transcriptional activity of AR and B-MYB, regulating genes such as *PLK1*, involved in cell cycle progression. Interestingly, the compound B3 showed antiproliferative effects in BC cell lines. Findings from this study suggest that the inhibitor specifically targets KDM4B in late S-phase due to activation of *PLK1* transcription *via* B-MYB, justifying the development of this KDM4B inhibitor for AR^+^ prostate cancer and opening up the possibility for new treatments in the AR^+^ subgroup of BC (93).

Other selective KDM family inhibitors are also described as anticancer agents in BC. Many reports indicate that KDM5 maintains tumor-initiating cells and promotes the development of drug tolerance (94). A selective inhibitor of KDM5B, KDOAM-21, significantly increased global levels of H3K4me3 in MCF-7 and TNBC cells. The compound also inhibited the growth of MCF-7 cells at 5 µM in colony-formation experiments (95). In another study, YUKA1, a small molecule inhibitor of KDM5A, displayed the ability to prevent drug tolerance in HER2^+^ BC cells treated with trastuzumab (96).

### Natural Inhibitors

Quercetin (WO2007104314) is a natural flavonoid that was found to inhibit KDM4C in demethylation assays and to modify H3K9me3 demethylation status in esophageal carcinoma and bone osteosarcoma cells. A hydroxamate analog (JP2011168581) showed selective inhibition of KDM4A/C (88). Methylstat (US20130137720) is a methyl ester analog inhibiting KDM4C that increases hypermethylation levels of H3K9me3 and H3K36me3 in a concentration-dependent manner, blocking the growth of MCF-7 cells (97).

Curcumin derivatives show good inhibition of KMD4s at cellular level. For example, efficient histone demethylation was observed by FLLL compounds. Notably, FLLL-8 and FLLL-24 displayed inhibitory activity against KDM4C, while FLLL-60 showed inhibition of KMD4A/D (98). Recently, a new compound synthesized from the natural product purpurogallin was reported to be a KDM4 inhibitor. This compound, called 9bf, exhibited a potent inhibitory activity on KDM4A and antiproliferative activity in many solid cancer cells (99).

### Peptide Inhibitors

In 2014, the first peptide-based KDM4 inhibitors displaying major selectivity and minor off-target effects were described (100). Two cyclic peptides were identified and both were active against KDM4C. Interestingly, this study proposed a novel approach to developing selective KDM4 inhibitors, regardless of the substrate and cofactor used (100).

An *in vitro* screening of a cyclic peptide library identified selective substrate-competitive inhibitors of KDM4s, showing alteration of H3K9me3 levels and inhibition of cell proliferation. The cyclic peptide CP2 showed potent IC_50_ values (IC_50_ = 0.42/0.33/0.39 μM against KDM4A/B/C, respectively) and exceptional intra-subfamily selectivity. The compound displayed high potency against KDM4A/B/C but was much less active against KDM4D (IC_50_ = 6.2 μM) and KDM4E (IC_50_ = 9.2 μM) (101). Although further studies are needed to evaluate stability, cell permeability, and subcellular localization, this approach may lead to the discovery and characterization of potent peptide inhibitors of KDM4 for the treatment of BC and other cancers. Because the functions of non-catalytic domains of KDM4 subfamily members such as PHD and Tudor domains are still unknown, the development of KDM4 inhibitors against non-catalytic domains remains challenging.

### Dual and Other Inhibitors

Since epigenetic machinery such as DNA methylation and histone modifications often work in parallel, the use of single agents in combination has recently drastically increased as this approach enhances their efficacy (102). Such a drug combination approach has also been exploited toward non-epigenetic targets (103). For example, combinations of HDAC-HSP90 inhibitors (104), HDAC-DNMT inhibitors (105), HDAC-KDM1 inhibitors (106), HDAC-BET protein inhibitors (107, 108), HDAC-EZH2 inhibitors (109), and HDAC-PI3K inhibitors (110) showed good efficacy in different cancer cells.

By way of an example, the dual KDM inhibitor MC3324 showed inhibition of KDM1 and KDM6A with a consequent increase in H3K4me2 and H3K24me3 levels and induction of apoptosis in hormone-responsive MCF-7 cells. Downregulation of ERα was observed at both transcriptional and translational level, indicating that the compound affects the transcription of genes regulating cell proliferation, hormonal response, and apoptosis. Interestingly, MC3324 reduced cell proliferation in *ex vivo* BC models and showed absence of toxicity and good oral efficacy in chicken embryo and mouse xenograft models. Thus, the simultaneous inhibition of multiple targets could be beneficial in BC (111).

Combining different drugs could be a feasible strategy to target multiple oncogenic pathways (112–114). Currently, many two-in-one drug approaches are being investigated in clinical trials for various cancers. The well-known HDAC inhibitor vorinostat (SAHA) in combination with tamoxifen (NCT00365599), and carboplatin and nab-paclitaxel (NCT00616967) is at different stages of clinical trials. In another trial, entinostat (MS-275) in combination with immunotherapy and monoclonal antibodies (nivolumab, ipilimumab) is under evaluation in patients with metastatic BC and HER2^-^ BC (NCT02453620). The synergy between HDAC inhibitors and anti-HER2 therapy with trastuzumab showed promising results (NCT00258349), but the adverse effects of trastuzumab resistance need to be further evaluated. In sum, in order to develop and optimize the effective use of epidrugs alone or in combination, there is an urgent need to identify new epigenetic targets that will pave the way for new cancer treatments.

In recent years, epigenetic studies combined with advanced computational methods have brought substantial advancements in drug discovery. A recent cutting-edge technology known as “epi-informatics” has been exploited to create a plethora of targeted compounds that may eventually lead to the discovery of new drugs. Computer-aided drug design could be used to explore and identify much needed selective KDM4 inhibitors for BC (115).

## Discussion

The role of KDM4s in cancer has been extensively studied, and promising targets for BC therapy have been proposed. Through demethylation of H3K9 and H3K36, KDM4s regulate chromatin structure and gene expression in numerous cancer types. Notably, overexpression of KDM4 subfamily members promotes cancer cell proliferation, invasion, and migration, DNA damage, tumor angiogenesis, and metastasis. Although some epigenetic mechanisms and functions of KDM4 proteins associated with carcinogenesis remain unclear, a growing body of evidence indicates that KDM4 inhibitors are good candidates as anticancer drugs for various malignancies, including BC. To date, however, reported inhibitors do not have a sufficient level of enzyme specificity and are not commercially available for the treatment of any cancer types. The specific role of KDM4s and their mechanism of action in BC is less well known. Another challenging task is to explore new compounds against KDM4 activity through computational screening, which may identify more specific KDM4 inhibitors. Further studies could drive the future development of potent and selective targets for specific KDM4s in BC.

To improve overall healthcare outcomes in BC, a substantial endeavor aimed at reducing mortality and increasing survival in patients is needed. Several studies have investigated the crucial role of KDM4 subfamily members in different cancers, and KDM4 targeting has been revealed as a promising strategy to inhibit BC development. However, no KDM4 modulators have as yet been approved for clinical use. Targeting these molecules has thus been attracting considerable interest among the scientific community (62, 88). The development of KDM4 inhibitors is still in its premature stage, with a limited number of scientific publications and patents. Although the development of potent and selective KDM4 inhibitors for BC is a complicated process, efforts in a number of different directions might be of help: i) The functions of KDM4E/F are still unclear, and more extensive investigations into these two enzymes may open up new avenues in cancer research. In addition, KDM4A/B/C share the same substrates, further complicating the development of selective inhibitors for KDM4 subtypes. Structural studies could help better define the catalytic pockets of these enzymes for more precise targeting (116, 117); ii) Specific gene expression patterns/programs controlling KDM4 activity are poorly studied and need to be further explored; iii) Findings related to KDM4 inhibitors usually derive from *in vitro* or cell-based assays, with a lower amount of *in vivo* data being reported. Characterizing their *in vivo* activity might provide greater insights useful for more potent drug development; iv) The activity of KDM4s in regulating DNA damage, non-histone proteins, and other posttranslational modifications is still unclear. New research directed at understanding these mechanisms may lead to the identification of novel molecules with higher selectivity: v) Due to their structural similarities and the presence of a JmjC domain in all isoforms of KDM4, engineering KDM4 inhibitors with isoform specificity is challenging. However, elucidating the distinct physiological function of each KDM4 enzymes in cancer is necessary.

Moving from a single- to a multi-KDM4s target therapeutic approach may be a useful strategy to improve BC treatment. Specifically, hybrid scaffolds coupling two individually well-known KDM4i compounds in a single unit (dual compound) could be a valid option to simultaneously target different KDM4 isoforms. As reported, the molecule MC3324 was more effective in blocking cell proliferation, targeting ER, and inducing cell death of BC cells, compared to its constituent moieties and other known inhibitors used alone or in combination. Alternatively, dual compounds could also be used to directly target KDM4 isoforms and their co-regulators in a highly specific manner. Hybrid scaffolds bridging binders of KDM4 isoform domains could be used to target a dual compound to KDM4 isoforms, thus overcoming the lack of specificity towards isoforms. However, the complexity and vulnerability of epigenetic regulation limits the use of epigenetic molecules to specific treatment contexts, which may contribute to poor therapeutic outcome. Studies into combinatorial epigenetic therapy have recently paved the way toward exploring new effective therapeutic strategy in cancer. For instance, polyclonal tumors are characterized by the presence of multiple coactive deregulated pathways, and in these tumors epigenetic alterations are favoring, permissive, or secondary events. In this scenario, testing novel targeted treatments in a single-agent approach may thus be problematic and may underestimate their effectiveness. Combining epigenetic drugs with conventional protocols, both targeted and immune therapies, may therefore represent a successful anticancer approach.

Exploiting single-cell omics approaches could capture cancer cell heterogeneity and provide a better understanding of the involvement of different KDM4s in the sequential stages of breast transformation at both bulk and single-cell level. This approach may ensure a more accurate patient stratification and unravel the role of each KDM4 in BC transformation, allowing evaluation of the efficacy of targeted selective modulators and opening the way toward personalized medicine in BC driven by specific KDM4 aberrations.

Unlike genetic events, epigenetic changes are reversible and because of this inherent plasticity, epigenome-targeted therapy has emerged as a potential strategy for the treatment of cancer. The results of investigational and approved epigenetic therapies in other clinical contexts have proven that this approach can be effective. KDM4s have been found to control many aspects of BC, including cancer initiation and progression. Additionally, traditional BC treatments fail in targeting therapy-resistant cancer stem cells strongly characterized by alteration of epi-regulators. Thus, considering that KDM4s are epigenetic regulators with overlapping functions in controlling gene expression of crucial signaling pathways, KDM4s inhibition reflects their target potential for BC therapy. Targeting these histone demethylases will pave the way toward improving the treatment of BC patients.

## Author Contributions

Conceptualization and writing BV, NDG, and GC. Supervision AN and LA. All authors contributed to the article and approved the submitted version.

## Funding

This work was supported by the Italian Association for Cancer Research (AIRC-17217), the Campania Regional Government “Lotta alle Patologie Oncologiche” iCURE (CUP B21C17000030007), MIUR Proof Of Concept (POC01_00043), VALERE: Vanvitelli per la Ricerca Program: AdipCare (ID263) and EPInhibitDRUGre (CUP B66J20000680005).

## Conflict of Interest

The authors declare that the research was conducted in the absence of any commercial or financial relationships that could be construed as a potential conflict of interest.

## Publisher’s Note

All claims expressed in this article are solely those of the authors and do not necessarily represent those of their affiliated organizations, or those of the publisher, the editors and the reviewers. Any product that may be evaluated in this article, or claim that may be made by its manufacturer, is not guaranteed or endorsed by the publisher.
